# A Portable, Single-Use, Paper-Based Microbial Fuel Cell Sensor for Rapid, On-Site Water Quality Monitoring

**DOI:** 10.3390/s19245452

**Published:** 2019-12-11

**Authors:** Jong Hyun Cho, Yang Gao, Seokheun Choi

**Affiliations:** Bioelectronics & Microsystems Laboratory, Department of Electrical & Computer Engineering, State University of New York-Binghamton, Binghamton, NY 13902, USA; jcho30@binghamton.edu (J.H.C.); ygao37@binghamton.edu (Y.G.)

**Keywords:** paper-based, microbial fuel cell, biosensor, water quality monitoring

## Abstract

Human access to safe water has become a major problem in many parts of the world as increasing human activities continue to spill contaminants into our water systems. To guarantee the protection of the public as well as the environment, a rapid and sensitive way to detect contaminants is required. In this work, a paper-based microbial fuel cell was developed to act as a portable, single-use, on-site water quality sensor. The sensor was fabricated by combining two layers of paper for a simple, low-cost, and disposable design. To facilitate the use of the sensor for on-site applications, the bacterial cells were pre-inoculated onto the device by air-drying. To eliminate any variations, the voltage generated by the microorganism before and after the air-drying process was measured and calculated as an inhibition ratio. Upon the addition of different formaldehyde concentrations (0%, 0.001%, 0.005%, and 0.02%), the inhibition ratios obtained were 5.9 ± 0.7%, 6.9 ± 0.7%, 8.2 ± 0.6%, and 10.6 ± 0.2%, respectively. The inhibition ratio showed a good linearity with the formaldehyde concentrations at R^2^ = 0.931. Our new sensor holds great promise in monitoring water quality as a portable, low-cost, and on-site sensor.

## 1. Introduction

Water quality monitoring is essential for providing clean and safe drinking water to the public as well as for ecological safety. However, in many parts of the developing world, this basic human need is neglected as efficient water treatment plans and infrastructures are lacking [1]. Developed countries are not exempt from this problem as a rapid increase in human activities, such as urbanization and industrialization, is responsible for severe water pollutions [2]. As such, providing access to clean water has become one of the greatest challenges in recent decades for both developing and developed countries.

Currently, the conventional methods used to detect water contaminants include chromatographic and spectroscopic methods, which require expensive equipment and highly trained personnel [3]. For the past few decades, numerous water toxicity biosensors have emerged utilizing enzymes, antibodies, and microorganisms, but these also suffer from the fact that, due to their specificity, they can only detect specific known chemicals, which would not be suitable for monitoring unexpected contaminants in water [4,5]. To maintain a safe water quality, there is an urgent need for a rapid and portable sensor for on-site and real-time measurements of toxic components in water.

Recently, microbial fuel cells (MFCs) have shown great potential as generic biosensors for monitoring toxic compounds in water [6,7,8]. MFCs utilize electrochemically active microorganisms to breakdown organic substrates to produce electrical energy. When these microorganisms are exposed to toxic compounds, their metabolic activities can be inhibited, thus decreasing their electron transfer reactions [9]. The changes in electrical outputs can be measured to determine the presence and intensity of toxins in water [7]. Compared to the conventional biosensors mentioned previously, MFC sensors have several advantages in that (i) they do not require an additional transducer or power source, (ii) monitored water samples provide the organic substrates necessary to microorganisms, (iii) the biofilm in the MFC provides self-repairing and self-sustainable properties, and (iv) microorganisms can replace expensive metal catalysts [10,11]. 

To further improve upon the traditional MFC sensors, we integrated paper-based electronics with the MFC sensor. Paper-based electronics, or “papertronics”, have recently attracted a lot of attention, as electronic and fluidic components can be printed onto a paper substrate [12]. Papers offer unique characteristics as electronic components of a device in that they are available in abundance, as well as being cost-effective, flexible, biodegradable, and disposable [13]. Previously, papers have been used in the fabrication of MFCs to provide power for small, portable transceivers and point-of-care diagnostic devices in remote areas [14,15,16]. Paper-based MFC sensors will not require expensive manufacturing materials or other external equipment such as pumps and tubes [17], which will make these sensors portable and a truly stand-alone, self-sustainable device.

**Figure 1:**
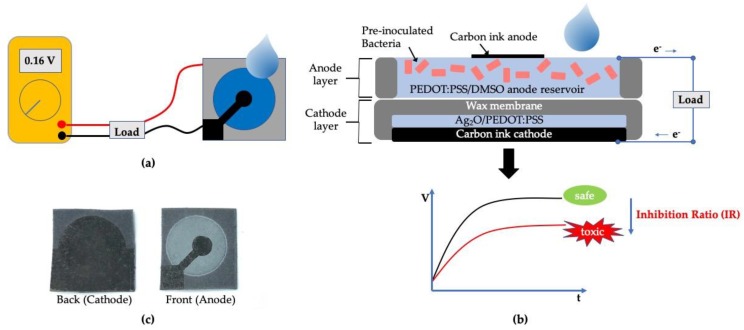
Schematic diagram of (**a**) the paper-based MFC sensor and (**b**) its cross section and working principle. (**c**) Photo images of the MFC sensor from the back and front side.

In this work, we demonstrated a novel paper-based, single-use MFC sensor for the rapid detection of toxins in water (Figure 1). The MFC sensor was created by combining two layers of paper for a low-cost, disposable, and simple structure. For the simplicity of testing, microorganisms were pre-inoculated onto the device and air-dried, making it easier for on-site measurements with a simple drop of water on the anode. The changes in the voltage can be measured with a small, low-cost multimeter. To compensate for any external factors and variations among different devices, the voltage of a device before and after air-drying was obtained and the differences were calculated as an inhibition ratio (Figure 1b). After the detection of the toxic compounds in water, the device was easily and safely disposed of by incineration to prevent environmental contamination.

## 2. Materials and Methods

### 2.1. Device Fabrication and Operating Principle

**Figure 2:**
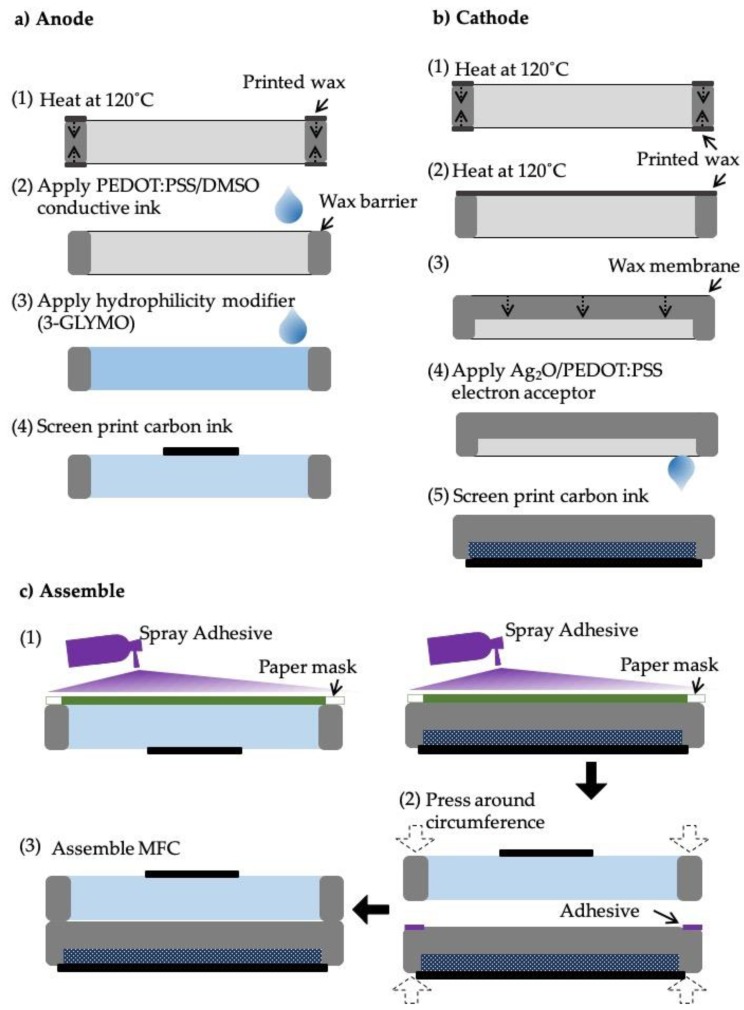
Fabrication steps of (**a**) anode layer and (**b**) cathode layer of the MFC sensor. (**c**) The assembly of the two layers into one device.

The MFC devices were fabricated onto Whatman #1 filter papers. The anode and cathode layers were prepared separately and later combined to form a 2-layer paper-based MFC. For each layer, the hydrophilic reservoir was defined by patterning wax around it to form a hydrophobic barrier. Wax patterns were designed using AutoCAD software and were printed onto the papers using a solid-wax printer (Xerox ColorQube 8570, Xerox, Norwalk, CT, USA). As seen in Figure 2, first the wax boundary patterns were printed on both sides of the anode and cathode papers and were heated at 120 °C for 2 min to allow the complete penetration of the wax into the papers. Then, on the cathode layer, a wax proton exchange membrane (PEM) was fabricated by printing wax on only one side and heating it at 120 °C for 40 s to allow only a partial penetration of the wax.

On the anode reservoir, a conductive ink consisting of 1wt% poly (3,4-ethylene dioxythiophene):poly(styrene sulfonate) (PEDOT:PSS) (Clevios PH1000, Heraeus, Vandalia, OH, USA) and 5wt% dimethyl sulfoxide (DMSO, Sigma-Aldrich) was injected and air-dried. Then, 2wt% 3-glycidoxypropy-trimethoxysilane (3-GLYMO) was pipetted to improve the hydrophilicity [13] (Figure 2a). For the cathode, a silver-based mixture was prepared by adding 100 mg of Ag_2_O to 2 mL of the conductive ink. Our previous works have demonstrated that, compared to the widely used potassium ferricyanide and air-cathode used in the preparation of cathodes in MFCs, Ag_2_O showed greater power performance and provided more versatile device design [13,18]. The mixture was sonicated and vortexed before pipetting it onto the cathode reservoir (Figure 2b). Then carbon ink was screen-printed on top of the anode and cathode reservoirs and was left to be air-dried overnight. Afterwards, the two layers were combined by spraying adhesives on the peripheries of the reservoirs [13] (Figure 2c).

Once the device was assembled, bacteria (125 μL) was inoculated on the anode surface and the performance of the MFC sensor was measured. The device was then air-dried at 30 °C for 3 h to let the bacterial media completely dry out. Then, different concentrations of the toxin in water samples were used to rehydrate the bacteria on the anode to measure the toxic shock effect. The change in the voltage, before and after drying the bacteria on the anode, was calculated and compared to different concentrations of the toxin and to the control (no toxin).

### 2.2. Microorganisms

Wild type *Shewanella oneidensis* MR-1 was obtained from −80 °C glycerol stock cultures and grown in a 15 mL Luria-broth (LB) medium with gentle shaking in air for 24 h at 30 °C. The LB medium consisted of 10.0 g tryptone, 5.0 g yeast extract, and 5.0 g NaCl per liter. The culture was then centrifuged at 4000 rpm for 10 min to remove the supernatant. The bacterial cells were then re-suspended in a new LB medium and used as a sensing microorganism for water toxicity monitoring. The media were autoclaved at 121 °C for 40 min prior to use.

### 2.3. Test. Setup and Analysis

The potentials between the anodes and cathodes were measured with a data acquisition system (NI, USB-6212) and were recorded via a customized LabView interface. An external resistor was used to close the circuit between the anode and the cathode. The current was calculated via Ohm’s law, I = V/R, and the power output was calculated via Joule’s law, P = V × I. Both the current and power densities were normalized to the anode area. To compensate for any external factors and variations among different devices, the changes in the voltage before and after the drying of the inoculum were calculated as an inhibition ratio (IR) to assess the sensitivity according to the following equation [10]:

(1) $$IR\left(\%\right)=\;\frac{V_{n}-\;V_{t}}{V_{n}}\times 100$$

where *V_n_* is the voltage before drying and *V_t_* is the voltage after drying when the device is rehydrated with a toxic solution.

To test the paper-based MFC sensor’s response to toxins, formaldehyde was used as a model toxic compound as it has been widely used for testing other MFC sensors [8,17]. The threshold limit of formaldehyde in water set by the EPA is 10 ppm (equal to 0.001% *v/v*) [19]. To demonstrate the toxic events, different concentrations of formaldehyde solutions were prepared in deionized water (0.001, 0.005, and 0.02% *v/v*). The formaldehyde solution was dropped onto the device after the air-drying of the bacterial inoculum in the anode, and the voltage was measured after 15 min of exposure.

### 2.4. Bacterial Fixation and Sanning Electron Microscope (SEM) Imaging

The paper-based MFCs were disassembled and the anodes were immersed in 5% glutaraldehyde for 2 h at 4 °C for bacterial fixation. Then, the samples were dehydrated by serial 15 min transfers through 35%, 50%, 75%, 95%, and 100% ethanol, followed by 10 min in hexamethyldisilazane (HMDS). The samples were air-dried overnight and were sputter-coated with carbon to be examined with a field emission SEM (Supra 55 VP, Zeiss, White Plains, NY, USA).

## 3. Results and Discussion

Microfluidic paper-based analytical devices (μPADs) have attracted a lot of attention as water quality sensors due to the easy fabrication and cost-effective nature of paper. The most commonly used method for μPAD-based water analysis is the colorimetric detection method [20,21,22]. However, colorimetric techniques can only provide qualitative or semi-quantitative analysis given that a calibration chart and external digital scanners or cameras are provided [23]. This approach also suffers from the possibility that the colored chemicals in the detection zones may not be uniformly distributed, leading to uneven and variable colors which can lower the sensitivity of the device [24]. Recently, electrochemical sensing with μPADs has grown considerably as it can provide quantitative data with a more rapid response and a higher sensitivity [23]. MFC sensors have additional advantages in that they do not require external power sources and only need a cheap multimeter to measure the voltage using two electrodes.

### 3.1. Operating Principle

In this work, an MFC sensor with two paper layers, an anode, and a cathode was used to test the level of the toxic compound, formaldehyde, in water. The non-conducting paper fibers were coated with a PEDOT:PSS polymer mixture, which formed a conductive reservoir that maintained the hydrophilicity without blocking the pores of the paper [25]. Once the bacteria were introduced, the hydrophilic, conductive anode reservoir quickly absorbed the bacteria-containing media via capillary action. The rapid absorption and inoculation of the bacteria onto the paper substrates allowed the MFC sensor to reach a stable open-circuit voltage (OCV) baseline in about 30 min.

**Figure 3:**
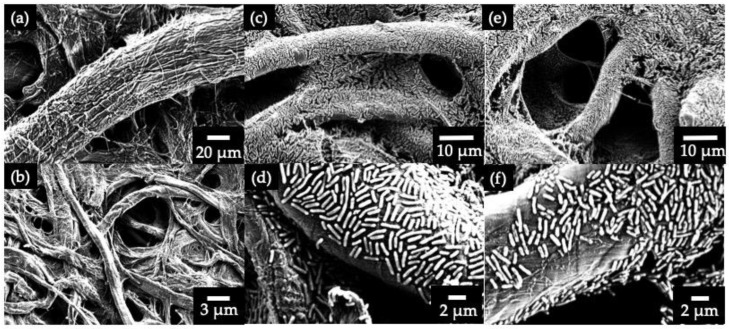
SEM images of the anode reservoirs. (**a**) and (**b**) are images of PEDOT:PSS coated anodes. (**c**) and (**d**) are images at 30 min bacterial inoculation. (**e**) and (**f**) are images at 3h bacterial inoculation.

Figure 3a,b shows the SEM images of the anode reservoir coated with PEDOT:PSS. Even after the PEDOT:PSS coating, distinct shapes of paper fibers and pores could still be observed, indicating that this polymer mixture was able to form a conductive reservoir without disrupting the paper matrix. Figure 3c,d shows the SEM images of the anodes 30 min after bacterial inoculation and Figure 3e,f shows 3 h after bacterial inoculation. Within 30 min of inoculation, very densely packed bacterial cells were observed throughout the porous 3D matrix of the paper. Typically, *S. oneidensis* MR-1 conducts extracellular electron transfer (EET) via two mechanisms: (i) direct electron transfer, where the cells are physically attached to the anode surface and (ii) indirect shuttle transfer, where the electrons are transferred to the anode via electron mediators. The distribution of bacteria in the paper matrix shows that the main EET mechanism of *S. oneidensis* MR-1 is based on direct electron transfer. The method of using electron mediators is unlikely, as all shuttling chemical compounds were removed by centrifugation before bacterial inoculation [12,26]. Even after 3 h, when the media were completely air-dried at 30 °C, the same number of densely packed bacterial cells was observed.

### 3.2. Formaldehyde Analysis

After 3 h of air-drying the bacterial media on the anode, the MFC sensors were tested to detect the formaldehyde concentrations. A 125 μL of a formaldehyde solution sample was directly dropped onto the anode, which enabled the rehydration of dry *S. oneidensis* MR-1 and dry nutrition into an active phase.

**Figure 4:**
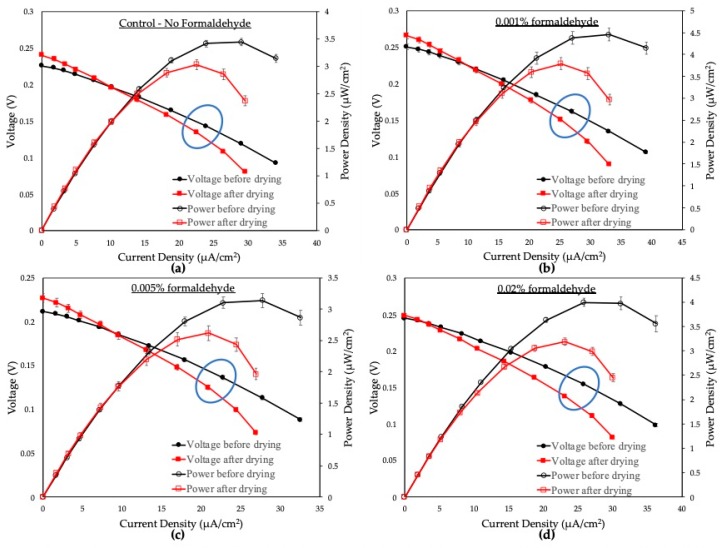
Polarization curves and power outputs of the devices with varying concentrations of formaldehyde. Black-circle lines and red-square lines represent before and after air-drying, respectively. (**a**) is the control with no formaldehyde; (**b**) 0.001%, (**c**) 0.005%, and (**d**) 0.02% formaldehyde. Blue circles represent voltages at 22.1kΩ resistor.

To measure the performances of the devices, a polarization curve and the power output of each MFC sensor were calculated from the saturated current values at different external resistors (470 kΩ, 249 kΩ, 162.8 kΩ, 100 kΩ, 71.3 kΩ, 47.5 kΩ, 32.2 kΩ, 22.1 kΩ, 15 kΩ, 10 kΩ). Figure 4 displays the polarization curves and power outputs of the MFC sensors with varying concentrations of formaldehyde (0%, 0.001%, 0.005%, 0.02%). Each test was performed in triplicate. From these graphs, we evaluated the internal resistances of the MFC sensors from the ohmic loss regions, where the curves showed a linear voltage drop. It is in good agreement that the internal resistance corresponds to the external resistor value, where the maximum power density is obtained [27]. For our MFC sensor, the maximum power output was obtained from a 22.1 kΩ external resistor, indicating that the internal resistance was also around 22.1 kΩ. Therefore, 22.1 kΩ was chosen as the optimal external load, and the corresponding voltages were used in the calculation of inhibition ratios according to Equation (1).

**Figure 5:**
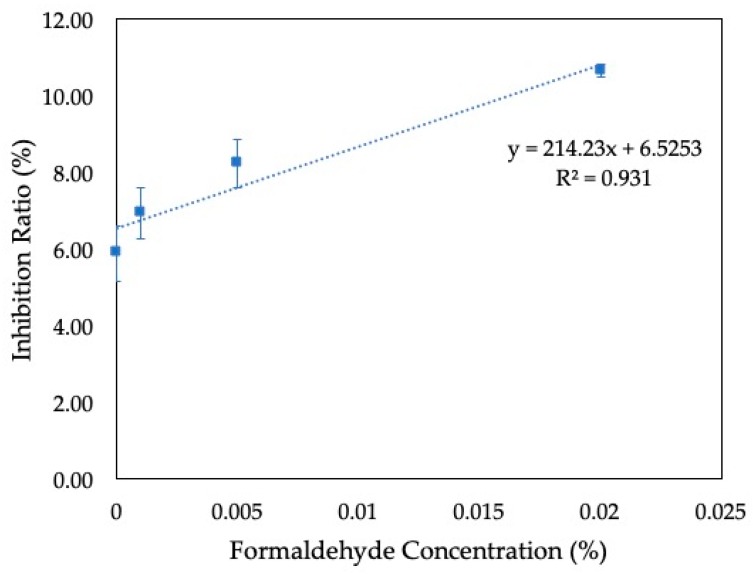
Calibration curve of the MFC sensor showing inhibition ratio (%) vs. the formaldehyde concentration (%). The curve shows good linearity with R^2^ = 0.931. The values are generated from a 22.1kΩ resistor and tests were performed 3 times at each concentration.

From Figure 4, blue circular areas represent the voltages of the MFC sensor before and after the drying of the bacteria using a 22.1 kΩ resistor. We can see that, upon the addition of the formaldehyde, the voltage decreased, showing inhibitory effects of the bacteria at different concentrations. The values of *V_n_*, *V_t_*, and IR with its standard deviation are summarized in Table 1. When a control medium with no formaldehyde was introduced to the anode, there was an inhibition of 5.9 ± 0.7%. This shows that even though most bacterial cells survived air-drying and dehydration, some cells were damaged in the process, decreasing the maximum voltage output. While most bacterial cells could withstand the effects of 3 h of air-drying, longer drying time, as well as storage conditions such as temperature and humidity, could negatively impact the bacteria’s viability. The effects of desiccation and long-term storage conditions are areas that require further studies in the future. The IR values for 0.001%, 0.005%, and 0.02% of formaldehyde were 6.9 ± 0.7%, 8.2 ± 0.6%, and 10.6 ± 0.2%, respectively. When the concentrations of formaldehyde and the IR were compared, the calibration curve of the device showed a good linearity at 0%–0.02% formaldehyde (R^2^ = 0.931) (Figure 5). Our results show a significant improvement compared to our previous work, which utilized a poly (methyl methacrylate) (PMMA) platform and external pumps to detect a formaldehyde concentration range of 0.003% to 0.075% [7]. The sensitivity of our device was also much better than the recently proposed paper-based MFC sensor from Chouler et al. that could only monitor the formaldehyde concentration at 0.1% [17].

With this work, bacteria were pre-inoculated onto the device and air-dried to facilitate the use of the MFC sensor for on-site applications. To show the sensitivity of the device, we followed the EPA threshold limit for formaldehyde in water, which is 10 ppm (0.001%). Our results show that even though the sensor could detect some changes in voltage at 0.001% formaldehyde, there is still an overlap of values with 0% formaldehyde. Therefore, the IR values comparing 0% and 0.001% formaldehyde did not show much statistical significance compared to other IR values at 0.005% and 0.02%. Accordingly, these results suggest that the MFC sensor is sensitive and suitable for the detection of formaldehyde down to 0.005%. When different formaldehyde concentrations were introduced to the dehydrated bacteria, there was a linear correlation to the inhibition ratios.

### 3.3. Disposal by Incineration

**Figure 6:**
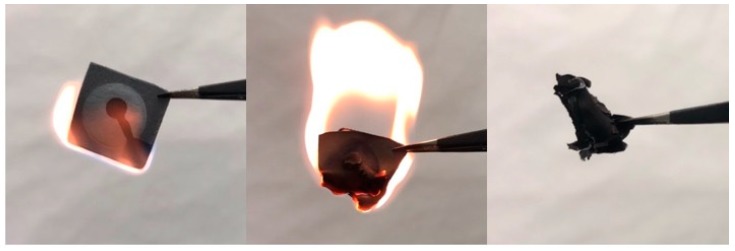
Disposal of the paper-based MFC sensor by burning. The device burned in about 30 s.

One of the requirements for portable, single-use devices is that they need to be easily disposable. If MFC devices are not carefully disposed of, the fabrication materials and microorganisms may contaminate the environment. Unlike many plastic-based devices and electronic wastes that require complicated procedures for disposal, paper-based devices can be easily and economically disposed of by simple burning. To reduce the negative impact on the environment, our MFC sensor was simply burned after use using a flame (Figure 6). The paper device took approximately 30 s to burn.

## 4. Conclusions

In this work, we demonstrated the feasibility of a portable, single-use, paper-based MFC sensor in detecting formaldehyde in water. The paper reservoirs were made conductive with PEDOT:PSS, and the hydrophilic zones were defined by patterning wax onto the paper. This new sensor utilized air-dried, pre-inoculated bacteria to detect different formaldehyde concentrations in water. This MFC sensor holds promise as a portable device that is capable of on-site measurements, as it is light, cheap, easy to use, and disposable. Our future work will include increasing the shelf-life of the MFC sensors with air-drying or freeze-drying techniques for long-term storage. Additionally, the generic biosensor with pre-inoculated bacteria may suffer from low levels or absence of substrates in the water, which can result in less or no current production. To overcome this problem, pre-loading organic substrates on the device with bacteria will also be studied. We will also work to make a portable and reusable voltage meter with an integrated readout LED that monitors the voltage of the biosensor.

## Figures and Tables

**Table 1:** *V_n_*, *V_t_*, IR, and standard deviation values from different formaldehyde concentrations.

Concentration (%)	V_n_ (V)	V_t_ (V)	IR (%)	Standard Deviation (V)
0	0.143	0.134	5.9	0.7
0.001	0.162	0.150	6.9	0.7
0.005	0.136	0.125	8.2	0.6
0.02	0.154	0.138	10.6	0.2
